# Diagnosis through differentiation: a pilot study on improving the diagnostic efficiency of primary headaches in ICHD3

**DOI:** 10.3389/fneur.2025.1727986

**Published:** 2025-12-18

**Authors:** Pengfei Zhang, Roger Cheng

**Affiliations:** 1 Department of Neurology, Beth Israel Deaconess Medical Center, Boston, MA, United States; 2 Department of Neurology, Harvard Medical School, Boston, MA, United States; 3 Department of Neurology, Rutgers Robert Wood Johnson Medical School, New Brunswick, NJ, United States

**Keywords:** headache classification, headache diagnosis, migraine, primary headache disorders, tension-type headache, ICHD3, computational medicine

## Abstract

**Background:**

What is the minimum number of questions necessary to diagnose any given primary headache disorder? The answer to this question may provide clinicians with a more efficient approach to history taking and lead to a more concise form of ICHD3. In this project, we attempt to address this problem mathematically.

**Methods:**

We defined a headache phenotype as a collection of characteristics, variables that can take on a Boolean (true/false) value and correspond to elements of the diagnostic criteria for each headache in ICHD3. There may be multiple phenotypes that fit a given diagnosis in the ICHD3. We extracted all characteristics used to describe primary headaches up to two levels deep in the hierarchy in the ICHD3. Episodic syndromes associated with migraine—recurrent gastrointestinal disturbance, benign paroxysmal vertigo, benign paroxysmal torticollis—were excluded because these require a primary diagnosis of migraine. For each headache diagnosis, we determined its “necessary true” (NT) characteristics. We also generated a list of “necessary false” (NF) characteristics by identifying characteristics that logically contradict the NT for each given diagnosis. We then sought to algorithmically identify the smallest set of NT and NF needed to differentiate between all primary headache diagnoses. As a result, any primary ICHD3 headache phenotype can be diagnosed once both the NT and NF criteria are satisfied. We verified this by translating all the possible conditions described by the ICHD3 criteria to our phenotype encoding schema.

**Results:**

We were able to minimize the NT and NF criteria to a set of 22 and 6 characteristics, respectively, with 5 overlaps between the groups. This results in a final set of 23 unique characteristics. Though an even smaller NT set may be possible, we are limited by computational power. These characteristics can be queried by using the following questions to generate a headache phenotype: (1) duration, (2) frequency, (3) sudden/rapid onset, (4) laterality, (5) clearly remembered onset, (6) sharp contour, (7) severity, (8) relationship to sleep/awakening, (9) reversibility of aura, (10) stabbing quality, and whether the headache can be triggered by (11) sex, (12) compression, (13) traction, (14) cold, or (15) exercise.

**Conclusion:**

Fifteen questions are necessary to differentiate the primary headache disorders in ICHD3. A smaller set may be possible, but we cannot prove its existence. Using this reduced set of questions, clinicians may be able to more efficiently arrive at ICHD3 diagnoses, but further research is required.

## Background

The International Classification of Headache Disorders (ICHD3) allows primary headaches to be diagnosed through standardized criteria (1, 2). By our estimate, there are more than 100 criteria for primary headache diagnoses (3). Although one may be able to assess multiple criteria concurrently with well posed clinical questions, the time required to comprehensively cover these criteria is significant, or worse yet can seem like an overwhelming task to trainees or non-experts in the subspecialty. This becomes more so in scenarios where multiple headache disorders co-exist. From a patient perspective, while a prospective headache diary is one of the best tools for headache diagnosis, the number of questions a patient might be asked to track could be construed as burdensome and could result in reduced compliance (4).

Instead of direct diagnosis of a given primary headache disorder using the full ICHD3 criteria, a potentially quicker alternative approach may be to simply differentiate one disorder from another. For example, although migraine without aura and episodic tension type headaches require 14 and 9 criteria/questions respectively for diagnosis, only 3 criteria (photophobia, phonophobia, and nausea) are needed to differentiate the two (1). As long as it can be established that a primary headache disorder exists, there may be a much smaller list of criteria that are already sufficient to arrive at the diagnosis.

This leads to the following question: What is the minimum number of questions required to uniquely identify a primary headache disorder once secondary headache has been ruled out? In this pilot study, we show that it is possible to use as few as 15 questions to differentiate primary headache disorders, up to two levels deep, in the ICHD3 hierarchy. Although this represents an efficient method of headache diagnosis, we will also point out the weaknesses and limitations of this approach.

## Methods

This study was done in 4 phases: (1) Data gathering and translation. (2) Generation and minimization of necessarily true (NT) criteria. (3) Generation and minimization of necessarily false (NF) criteria. (4) Algorithmic proof and verification of the result.

### Phase 1: inclusion criteria, data gathering, and translation phase

We included all primary headache disorders up to two levels deep in ICHD3. Since we are only concerned with the diagnosis of primary headache disorders and not their complications, subsections 1.3—“chronic migraine,” 1.4—“complications of migraine,” and 1.6—“episodic syndrome that may be associated with migraine” were excluded as they require a primary diagnosis of migraine. Diagnoses of “probable” headaches were also excluded.

To manipulate the included ICHD3 disorders as numerical data, we used prime number encoding, a method previously published for evaluation of concurrent diagnosis in ICHD3 (3). We will briefly describe this encoding process but refer interested readers to the original article.

First, we defined a headache *phenotype* as a collection of *characteristics*; *characteristics* are variables that can take on a Boolean (true/false) value. There may be multiple *phenotypes* each consisting of a different set of characteristics that fit a given diagnosis in the ICHD3. The prime number encoding process involves translating phenotypes of the ICHD3 criteria into Boolean logical statements then assigning each a composite number.

For example, consider the criteria for migraine without aura (1):

A. *At least five attacks fulfilling criteria B–D*.
B. *Headache attacks lasting 4–72 hours (when untreated or unsuccessfully treated)*.
C. *Headache has at least two of the following four characteristics:*
  1. *unilateral location*,
  2. *pulsating quality*,
  3. *moderate or severe pain intensity*,
  4. *aggravation by or causing avoidance of routine physical activity (e.g. walking or climbing stairs)*,
D. *During headache at least one of the following:*
  1. *nausea and/or vomiting*,
  2. *photophobia and phonophobia*,
E. *Not better accounted for by another ICHD-3 diagnosis*.

Here, the descriptions of “unilateral location,” “photophobia,” “phonophobia,” and “pulsating” are *characteristics* since these variables can take on the value of either true or false. Now, take a patient with headache that is unilateral, pulsating, mild in intensity, not aggravated by physical activity, accompanied by nausea, and lasting 4–72 h. This headache profile satisfies the diagnosis for migraine without aura; therefore, the set of characteristics that make up this profile would be considered a *phenotype* of migraine without aura. Of course, it is not the only example of migraine without aura—therefore, a diagnosis in the ICHD3 (such as migraine without aura) will often consist of a set of multiple *phenotypes*.

The above criteria can be represented by the following logical statement using alphanumeric symbols for each characteristic:


$$\begin{array}{l} \text{A}\wedge \text{B}\wedge [(\text{C1}\wedge \text{C2})\vee (\text{C1}\wedge \text{C3})\vee (\text{C1}\wedge \text{C4})\vee (\text{C2}\wedge \text{C3})\vee (\text{C2}\wedge \text{C4})\vee \\ (\text{C3}\wedge \text{C4})]\vee (\text{D1}\wedge \text{D2})\wedge \text{E} \end{array}$$

The symbol “∧” represents the Boolean “AND” and “∨” represents “OR”. This logic statement—and indeed any logic statement—can be translated into a series of statements connected by an AND operator. This is called disjunctive normal form (5). That is, “…it is a disjunction (sequence of ORs) consisting of one or more disjuncts, each of which is a conjunction (AND) of one or more literals…” (6). Furthermore, every statement in logic can be put into this format as long as it consists of a combination of AND, OR, and NOT statements. (6) In the example above, the statement can be translated into:


$$\begin{array}{l} \left(\text{A}\wedge \text{B}\wedge \text{C}1\wedge \text{C}2\wedge \text{D}1\wedge \text{E}\right)\vee \left(\text{A}\wedge \text{B}\wedge \text{C}1\wedge \text{C}2\wedge \text{D}2\wedge \text{E}\right)\vee \\ \left(\text{A}\wedge \text{B}\wedge \text{C}1\wedge \text{C}3\wedge \text{D}1\wedge \text{E}\right)\ldots \end{array}$$

Given the disjunctive normal form, every characteristic is then assigned an arbitrary prime number. To represent a phenotype, the corresponding prime numbers for all characteristics that are present (true) are multiplied together to create a composite number representing the phenotype. Due to the unique mathematical property of a prime having no factors other than itself, logical values of phenotypes can be quickly compared to those of characteristics. By dividing the composite number representing a phenotype by the prime number encoding a characteristic, the remainder will be zero if and only if the composite number is a factor of the prime (that is, if the phenotype contains that characteristic). In the example above, each of the characteristics (A, B, C1, C2,… etc.) are assigned a prime number, and the above statement is then translated into a composite number through multiplication of its disjunctive normal form.

To preserve the original ICHD3 criteria without introducing editorial comment, we attempted to denote each individual characteristic verbatim (if possible) in our Boolean conversion. For example, the characteristics “4–72 h” (c.f. migraine without aura) and “30 min to 7 days” (c.f. tension type headache) were not merged despite overlapping in duration but are rather considered separate characteristics. Negative characteristics were also defined independently from their associated positive correlate—for example, “nausea and/or vomiting” (c.f. migraine without aura) and “no nausea and/or vomiting” (c.f. tension type headache) were considered separate characteristics. This was necessary since it allows for distinction between required absent—such as in tension type headache where the absence of nausea is required—and incidentally absent characteristics—such as in cases like nummular headache, where the absence of nausea has no bearing on the diagnosis.

Once all diagnoses in the included ICHD3 were converted into logic statements, duplicates were removed to create a single, comprehensive list of non-overlapping characteristics, which in various unique combinations, could be used to describe all possible primary headache disorders covered in this project.

### Phase 2: generation and minimization of necessary true (NT) characteristics

First, observe that while multiple phenotypes may result in migraine without aura as a diagnosis, these phenotypes share a core group of characteristics. For example, criteria B of the ICHD3 demands that all migraine without aura phenotypes contain the characteristic “attacks lasting 4–72 h.” We call this characteristic a “necessary true” (NT) characteristic for migraine without aura. While the exact combination of NT characteristics is unique for each individual primary headache diagnosis in the ICHD3, diagnoses may share similar NT characteristics. For example, hypnic headache and hemicrania continua share the same NT characteristic of having occurred for “greater than 3 months.” Because our goal in this project was to reduce redundancy, the question becomes: what is the minimum set of NT characteristics needed to uniquely identify each disorder?

To tackle this problem, we enumerated a comprehensive list of NT characteristics for each of the included diagnosis. We then designed an algorithm that generated all valid combinations of characteristics from the NT list resulting in a primary headache diagnosis in ICHD3. Next, characteristics were iteratively removed to determine whether the resulting combinations still uniquely resolved each diagnosis. This process continued until we arrived at the minimum number of criteria needed such that no two diagnoses shared the same combination of NT characteristics. To eliminate duplicate diagnoses, we mandated that combinations could not be subsets of each other.

### Generation and minimization of necessary false (NF) characteristics

It is not sufficient to obtain only a list of NT characteristics for all ICHD3 diagnoses, as one can imagine a phenotype having characteristic(s) which satisfy the NT characteristics for more than one distinct diagnosis. As a result, we must also design a strategy of differentiating between two given diagnoses when given a specific headache phenotype.

We addressed this by also obtaining the “necessary false” (NF) characteristics for each diagnosis. These are the characteristics that must not be true within a phenotype for that phenotype to still fit the diagnosis of a specific disorder. For example, since migraine without aura requires that the attacks be between “4–72 h,” migraine without aura therefore cannot last only “1–600 s” (c.f. SUN). Therefore, the characteristic “1–600 s” is a NF characteristic for migraine without aura.

As such, we generated a list of NF characteristics for individual headache diagnoses manually by noting all possible contradictory characteristics. As with the original list of NT characteristics, not all NF characteristics are needed to distinguish between any two given diagnoses. Therefore, the minimum set of NF characteristics needed such that no two diagnoses share the exact same combination can be determined using a similar algorithm to that previously used to minimize the list of NT characteristics. Of note, this search algorithm may result in combinations where one may be a subset of another yet still be considered unique. (Exclusion is unlike inclusion and subset does not lead to dual diagnoses.)

### Algorithmic proof

We now must verify that combinations of NF or NT pairings can differentiate between ICHD3 defined headache disorders. In other words, we seek to show that given any ICHD3 phenotype, its corresponding NT/NF pairing yields the same diagnosis. Once we derived the minimum set of NF and NT, we prove this algorithmically as follows.

First, note that in phase 1 of the project we generated a comprehensive list of phenotypes corresponding to all included primary headache disorders in ICHD3. We therefore simply need to show that each phenotype in this set can be mapped to the same corresponding primary headache diagnosis using our framework NF/NT framework. In the proof stage, we implemented a Haskell algorithm to do this comparison.

### Instrumentation

The search algorithms for all above procedures were implemented with custom code in the Haskell programming language using the Glasgow Haskell Compiler version 9.4.8. Of note, this project shares a shared core dataset with a prior paper (3).

## Results

### Minimization of necessary true (NT) characteristics

The list of primary headache diagnoses as well as their NT characteristics in their corresponding prime encoding form is listed in Supplementary material 1. Due to the combinatoric complexity and limitations in our computational power, our brute force algorithm cannot calculate a definitive minimum combination possible to differentiate all NT. However, approximate minimization can be arrived at by directing the search manually. We were able to uniquely differentiate all primary headache disorders with as low as 22 characteristics. These characteristics are, in their prime encoding: 3, 5, 7, 11, 17, 23, 67, 71, 83, 97, 101, 131, 139, 199, 223, 433, 439, 443, 487, 491, 503, and 523. How these characteristics uniquely differentiate NT for headache disorders is presented in Supplementary Table 1. A list of prime encodings and their represented characteristics is presented in Supplementary material 3.

### Minimization of NF

We manually generated the NF characteristics for each headache disorder. The results are presented in Supplementary material 2. By using NF characteristics in combination, it is not possible to differentiate between “hemicrania continua” vs. NDPH– this is because the NF is the same for both. Fortunately, this did not appear to affect our result given that their NTs are different. Furthermore, it is also impossible to exclude “cold induced headache,” “compression induced headache,” “nummular headache,” and “primary traction headache” through the NF strategy as these disorders do not contain criteria that are NF criteria. Despite the shortcoming, this likewise does not appear to affect the outcome.

We can determine computationally that 6 NF characteristics are sufficient to distinguish between the above diagnoses. These 6 characteristics are 5, 7, 23, 97, 199, and 547. We were able to algorithmically verify that further reducing this to 5 characteristic combinations would not be sufficient to satisfy our criteria, therefore proving that the characteristics above are a minimum result. The resultant NF for each headache is shown in Supplementary Table 2.

By combining the minimum NT and NF phases, we obtained the phenotypes in Supplementary Table 3. Given that 5 of the 6 NF are already present in NT, we obtained a final list of 23 characteristics necessary in differentiating primary headaches. These characteristics are 3, 5, 7, 11, 17, 23, 67, 71, 83, 97, 101, 131, 139, 199, 223, 433, 439, 443, 487, 491, 503, 523, 547. These characteristics correspond to 15 questions that can be queried regarding a patient's headache phenotype: (1) duration, (2) frequency, (3) sudden/rapid onset, (4) laterality, (5) clearly remembered onset, (6) sharp contour, (7) severity, (8) relationship to sleep/awakening, (9) reversibility of aura, (10) stabbing quality, and whether the headache can be triggered by (11) sex, (12) compression, (13) traction, (14) cold, or (15) exercise.

### Algorithmic proof

To verify our calculation, all phenotypes represented by the original ICHD3 were encoded and mapped to a diagnosis via our NT/NF system and compared with the corresponding original. We found that this list corresponds directly with our new set of characteristics (see Supplementary material entitled “Algorithmic Proof”).

## Discussion

Physicians' face to face time with patients is one of the most important resources in clinical practices (7, 8). Although the ICHD3 offers a scientific way to diagnose headache disorders, the number of questions required to make a primary headache diagnosis may be overwhelming, especially for non-specialists. This project seeks to improve clinical efficiency in the diagnosis of ICHD3 primary headache disorders without sacrificing accuracy via derivation of a more concise list of criteria which rely on differentiation rather than direct diagnosis.

Although our method is mathematical, we believe that clinicians should be able to verify our result by simply inspecting Supplementary Table 3. Indeed, the underlying principle of this project's methodology should be intuitively familiar to clinicians—the necessarily true (NT) conditions are what clinicians would call “pertinent positives” whereas the necessary false (NF) conditions are simply the “pertinent negatives.” The combination of these lists is what we intuitively do as clinicians to arrive at any diagnosis.

Headache duration appears to be the most important characteristic used to exclude certain diagnoses according to our results. This is likely because most primary headache disorders are characterized by duration of the symptoms and as such can be excluded by duration (for example, it is impossible for cluster headaches to be constant in the ICHD3). It is important to note that our analysis is only possible when duration is obtained as a range rather than a single number. For example: a 4-h long headache can fit both the “4–72 h” time characteristic for migraine without aura as well as the “30 min to 7 days” characteristic of tension-type headaches; knowing that a patient has a headache range of 4 to 8 hours and never only for 30 min will allow for exclusion of tension-type headaches. This is the reason why photophobia, phonophobia, and nausea—those features we often associate with migraine—are not necessarily in our algorithm for differentiation.

The reliance on duration is also an important limitation to our study. Taken at face value, “4–72 h” becomes a defining and unique characteristic of migraine without aura in the ICHD3—indeed the phrase “4–72 h” appears nowhere in ICHD3 except in the criteria/description of migraine without aura. (The same can be said for “30 min to 7 days” as the definitional duration of various types of tension type headaches.) It therefore makes sense that in our algorithmic approach this characteristic becomes vital and sufficient in our model. (Indeed, even phonophobia, photophobia, or nausea occur more frequently in the ICHD3 than this particular duration.) Although our method in this paper is to take ICHD3 literally without editorialization, we should comment that duration tends not to be the most defining feature in clinical practice; as ICHD3 itself admits that children can have migraine headaches as short as 2 h but yet never elevate this to the status of a “criteria.” Chronic migraine may be near constant in clinical practice, yet 72 h is nevertheless the definitional limit (1). As a result, when our method suggests a diagnosis where the criteria involves duration, we recommend clinicians take additional steps to verify such a diagnosis by interrogating other facets of the clinical history.

Several headache disorders cannot be diagnosed by exclusion: For example, primary sex headaches, primary thunderclap headache, and primary cough headache, can only be diagnosed by directly asking the patient about triggering characteristics. Counterintuitively, some of the criteria one most strongly associates with headache disorders do not seem to contribute toward differentiation. For example, autonomic features are not actually needed in arriving at the diagnosis of a trigeminal autonomic cephalalgia if laterality, the range of duration, and other characteristics can be accurately obtained. In fact, the inclusion of “restlessness” as one of the questions only really serves to exclude primary exercise headaches in our paradigm. We do not advocate the wholesale elimination of considerations for autonomic features in headache research/definition. Indeed, we believe that this to be a weakness in our study result: “proving” that a headache is a specific disorder is a different mode of inquiry than differentiating a specific headache from others. The existence of “probable” headache diagnoses in the ICHD3 highlights this point. We therefore advise that clinicians should ask patients clarifying questions if using our results in clinical practice.

Indeed, our decision to exclude probable headache disorders from our study is intentional: It is our assumption that in cases of a suspected primary headache disorder, a more comprehensive set of questions would be required to assess for and rule out alternative etiologies. In other words, it is our belief that if someone were to be diagnosed with probable migraine, then we should not be taking the NF/NT shortcuts to justify the diagnosis. Indeed, we must note that we did not show—and it is not our intention to show—that any headaches satisfying NT/NF criteria automatically satisfies ICHD3 criteria. (This is clearly impossible since our goal is to use a truncated set of characteristics as compared to ICHD3.)

Similarly, since our NF/NT shortcut decreases the number of questions required to make a diagnosis, our algorithm maybe unable to reach a diagnosis for patients who cannot recall specific characteristics of their headaches in detail. This is therefore a potential weakness of the study. To ameliorate this drawback, we recommend clinicians instruct patients to prospectively track relevant NF/NT characteristics if there is a problem with patient recall.

Furthermore, our paradigm operates on the assumption that a patient has ICHD3 primary headache disorder. (We therefore recommend using our paradigm only after screening for secondary headaches for red flags). If not, then our truncated method would not be sufficient to diagnose it. While this may appear to be obvious at first glance, in clinical practice, we expect that this would be much more complicated to recognize.

Finally, due to the combinatoric complexity of the NT problem, we are unable to algorithmically arrive at a minimum solution. As a result, we are not able to determine whether 22 characteristics (i.e., 1 less characteristic than our proposed 23) are sufficient to uniquely differentiate all our primary headache diagnoses: evaluating a combination of 22 from 52 elements amounts to testing for 270,533,919,634,160 combinations. Due to the limitations of computational power, we are not able to prove this as impossible. Therefore, a lower number of characteristics may be potential solutions to our problem.

## Conclusion

We mathematically derived a smaller set of phenotypes that can differentiate and diagnose primary headache disorders in ICHD3, resulting in an essential list of 23 which can be queried using 15 questions, though the question of whether an even simpler solution exists remains an open question due to computational limitations. We believe our list here represents an initial pilot attempt at simplification of headache diagnoses. Using this smaller set of phenotypes, clinicians may be able to more efficiently arrive at a headache diagnoses—however, as there are potential pitfalls to using only differentiation rather than confirming a diagnosis (as described above), we advise that this method should only be used for screening. Nevertheless, our study provides clues as to why some historical questions are more important than others with regard to headache diagnosis. Further research is required to overcome the limitations of this approach.

## Data Availability

The original contributions presented in the study are included in the article/Supplementary material, further inquiries can be directed to the corresponding author.
