# Supplementary material for: Diagnosis through differentiation: a pilot study on improving the diagnostic efficiency of primary headaches in ICHD3
Source: Front Neurol. 2025 Dec 18;16:1727986. doi: 10.3389/fneur.2025.1727986 (PMC12756135; doi:10.3389/fneur.2025.1727986)
Supplement: Supplementary file 1 [file Table_1.docx]

Table 1:

| Migraine without aura | 23 |  |
| --- | --- | --- |
| Migraine with aura | 131 |  |
| Infrequent Tension Type Headache | 199 |  |
| Frequent Tension Type Headache | 3 |  |
| Chronic Tension Type Headache | 139 |  |
| Cluster Headache | 11 | 523 |
| Paroxysmal Hemicrania | 17 | 523 |
| Short-lasting unilateral neuralgiform headache attacks | 5 | 523 |
| Hemicrania Continua | 97 | 523 |
| Primary Cough Headache | 503 |  |
| Exercise Headache | 67 |  |
| Primary Sex Headache | 71 |  |
| Thunderclap Headache | 223 |  |
| Cold Induced Headache | 443 |  |
| Compression Headache | 433 |  |
| Traction Headache | 439 |  |
| Primary Stabbing Headache | 491 |  |
| Nummular Headache | 487 |  |
| Hypnic Headache | 7 | 101 |
| New Daily Persistent Headache | 83 | 97 |
