# Supplementary material for: Diagnosis through differentiation: a pilot study on improving the diagnostic efficiency of primary headaches in ICHD3
Source: Front Neurol. 2025 Dec 18;16:1727986. doi: 10.3389/fneur.2025.1727986 (PMC12756135; doi:10.3389/fneur.2025.1727986)
Supplement: Supplementary file 2 [file Table_2.docx]

Table 2

| Migraine without aura | 5 | 97 | 7 |  |  |
| --- | --- | --- | --- | --- | --- |
| Infrequent Tension Type Headache | 97 | 5 |  |  |  |
| Frequent Tension Type Headache | 5 | 97 | 199 |  |  |
| Chronic Tension Type Headache | 5 | 199 |  |  |  |
| Cluster Headache | 5 | 23 | 97 | 547 |  |
| Paroxysmal Hemicrania | 23 | 97 | 547 |  |  |
| Short-lasting unilateral neuralgiform headache attacks | 7 | 23 | 199 | 97 | 547 |
| Hemicrania Continua | 5 | 7 | 23 | 199 | 547 |
| Primary Cough Headache | 23 | 97 |  |  |  |
| Exercise Headache | 7 | 97 |  |  |  |
| Primary Sex Headache | 97 |  |  |  |  |
| Thunderclap Headache | 547 |  |  |  |  |
| Primary Stabbing Headache | 7 | 23 | 547 | 97 |  |
| Hypnic Headache | 5 | 23 | 97 |  |  |
| New Daily Persistent Headache | 5 | 7 | 23 | 199 | 547 |
