# Supplementary material for: Diagnosis through differentiation: a pilot study on improving the diagnostic efficiency of primary headaches in ICHD3
Source: Front Neurol. 2025 Dec 18;16:1727986. doi: 10.3389/fneur.2025.1727986 (PMC12756135; doi:10.3389/fneur.2025.1727986)
Supplement: Supplementary file 3 [file Table_3.docx]

Table 3: Criteria sufficient to diagnose ICHD3 Primary Headache Disorders (bold represents necessary true, italics represents necessary false)

| Migraine without aura | **23** | *5* | *97* | *7* |  |  |  |
| --- | --- | --- | --- | --- | --- | --- | --- |
| Migraine with aura | **131** |  |  |  |  |  |  |
| Infrequent TTH | **199** | *5* | *97* |  |  |  |  |
| Frequent TTH | **3** | *5* | *97* | *199* |  |  |  |
| Chronic TTH | **139** | *5* | *199* |  |  |  |  |
| Cluster Headache | **11** | **523** | *5* | *23* | *97* | *547* |  |
| Paroxysmal Hemicrania | **17** | **523** | *23* | *97* | *547* |  |  |
| SUN | **5** | **523** | *7* | *23* | *199* | *97* | *547* |
| Hemicrania Continua | **97** | **523** | *5* | *7* | *23* | *199* | *547* |
| Primary cough headache | **503** | *23* | *97* |  |  |  |  |
| Exercise Headache | **67** | *7* | *97* |  |  |  |  |
| Primary Sex Headache | **71** | *97* |  |  |  |  |  |
| Thunderclap Headache | **223** | *547* |  |  |  |  |  |
| Cold Induced Headache | **443** |  |  |  |  |  |  |
| Compression Headache | **433** |  |  |  |  |  |  |
| Traction Headache | **439** |  |  |  |  |  |  |
| Primary Stabbing | **491** | *7* | *97* | *23* | *547* |  |  |
| Nummular Headache | **487** |  |  |  |  |  |  |
| Hypnic Headache | **101** | **7** | *5* | *23* | *97* |  |  |
| New Daily Persistent Headache | **97** | **83** | *5* | *7* | *23* | *199* | *547* |

Key:

*3 = 1 to 14 days per month, 5 = 1 to 600 seconds, 7 = 15 min up to 4 hours after waking, 11 = 15 to 180 minutes, 17 = 2 to 30 minutes*, *23 = 4 to 72 hours*, 67 = brought on by exercise, *71 = brought on by sex*, 83 = clearly remembered onset, *97 = constant*, *101 = developing only during sleep and causing wakening*, *131 = fully reversible aura*, *139 = greater than 15 days per month*, *199 = less than 12 days per year,* *223 = max within 1 minute,* *433 = resolve within 1 hour after removal of compression, 439 = resolve within 1 hour after removal of traction,* *443 = resolve within 30 min after removal of cold,* *487 = sharply contoured, 491 = single or series of stabs, 503 = sudden,* *523 = unilateral*, *547 = up to 72 hours with mild headaches*
