# Supplementary material for: Diagnosis through differentiation: a pilot study on improving the diagnostic efficiency of primary headaches in ICHD3
Source: Front Neurol. 2025 Dec 18;16:1727986. doi: 10.3389/fneur.2025.1727986 (PMC12756135; doi:10.3389/fneur.2025.1727986)
Supplement: Supplementary file 4 [file Data_Sheet_1.pdf]

migraine w/o aura:
5,7,11,17,47,97,541,557,
ndph:
2,3,5,7,11,17,19,23,47,101,103,107,131,179,193,199,211,433,439,443,547
,557,
exercise:
7,449,541,97,
thunderclap:
547,557,
cluster:
5,23,97,541,557,547,
ph:
23,97,179,541,557,547,
sun:
3,7,11,19,23,107,199,163,97,179,541,547,
hc:
2,3,5,7,11,17,19,23,47,101,103,107,131,179,193,199,211,433,439,443,547
,557,
mwa:
541,
stabbing:
2,7,11,17,19,23,179,547,541,97,
hypnic:
5,23,97,139,449,541,557,
cough:
23,97,541,
itth:
5,17,97,107,137,139,167,251,541,557,269,
ftth:
5,17,97,107,137,139,167,199,251,541,557,
ctth:
5,17,199,
sex:
97,577,541,
