# Supplementary material for: Diagnosis through differentiation: a pilot study on improving the diagnostic efficiency of primary headaches in ICHD3
Source: Front Neurol. 2025 Dec 18;16:1727986. doi: 10.3389/fneur.2025.1727986 (PMC12756135; doi:10.3389/fneur.2025.1727986)
Supplement: Supplementary file 5 [file Data_Sheet_2.pdf]

2,1 minute to 72 hours with severe
3,1 to 14 days per month
5,1 to 600 seconds
7,15 minutes up to four hours after waking
11,15 to 180 minutes
13,1–6 cm in diameter
17,2 to 30 minutes
19,30 min to 7 days in duration
23,4 to 72 hours
29,abrupt explosive intensity just before or with orgasm
31,aggravated by physical activity
37,at least one aura symptom is positive
41,at least one aura symptom is unilateral
43,at least one aura symptom spreads gradually over 5 minutes
47,between 1 second to 2 hours
53,bilateral location
59,brainstem aura
61,brought on by cold stimuli
67,brought on by exercise
71,brought on by sex
73,brought on within 1 hour of compression
79,brought on within 1 hour of traction
83,clearly remembered onset
89,conjunctival injection
97,constant
101,developing only during sleep and causing wakening
103,each individual aura symptom lasts 5–60 minutes
107,every other day to 8 per day
109,eyelid edema
113,fixed in size and shape
127,forehead and facial sweating
131,fully reversible
137,greater than 1 per day
139,greater than 15 days per month
149,greater than 2 episodes
151,greater than 20 episodes
157,greater than 5 episodes
163,greater than 5 minutes
167,greater than 5 per day
173,greater than 8 days per month
179,hours to days
181,increasing in intensity with increasing sexual excitement
191,indomethacin responsive
193,irregular frequency
197,lacrimation
199,less than 12 days per year
211,less than 48 hours
223,max within 1 minute
227,maximal at site of compression
229,maximal at site of traction

233,mild to moderate pain
239,miosis
241,moderate to severe
251,more than 1 episode per day
257,more than 10 episodes
263,more than 3 months
269,more than 10 days per month
271,motor aura
277,nasal congestion
281,nausea/vomiting
283,no conjunctival injection
293,no eyelid edema
307,no forehead and facial sweating
311,no lacrimation
313,no miosis
317,no nasal congestion
331,no nausea/vomiting
337,no orbital or supraorbital or temporal pain
347,no phonophobia
349,no photophobia
353,no ptosis
359,no restless
367,no rhinorrhea
373,nonpulsating
379,not aggrevated by activity
383,orbital or supraorbital or temporal pain
389,phonophobia
397,photophobia
401,provoke by cough
409,provoke by valsalva
419,ptosis
421,pulsating
431,relieve by triptan or ergot
433,resolve within 1 hour after removal of compression
439,resolve within 1 hour after removal of traction
443,resolve within 30 min after removal of cold
449,restless
457,retinal aura
461,rhinorrhea
463,round or elliptical
467,sensory aura
479,severe
487,sharply contoured
491,single or series of stabs
499,speech and/or language aura
503,sudden
509,the aura is accompanied, or followed within 60 minutes, by headache
521,two or more aura symptoms occur in succession
523,unilateral

541,unremitting within 24 hours
547,up to 72 hours with mild
557,up to few seconds
563,visual aura
