# Supplementary material for: Diagnosis through differentiation: a pilot study on improving the diagnostic efficiency of primary headaches in ICHD3
Source: Front Neurol. 2025 Dec 18;16:1727986. doi: 10.3389/fneur.2025.1727986 (PMC12756135; doi:10.3389/fneur.2025.1727986)
Supplement: Supplementary file 6 [file Data_Sheet_3.pdf]

Algorithmic Proof: Each line represents a numerical encoding of the original ICHD3 diagnosis as well as which diagnosis in the new schema that encoding maps to.

7580756461, migraine w/o aura maps to mwoa
13242732241, migraine w/o aura maps to mwoa
16451185183, migraine w/o aura maps to mwoa
102951563551, migraine w/o aura maps to mwoa
127894697713, migraine w/o aura maps to mwoa
223417708453, migraine w/o aura maps to mwoa
3191498470081, migraine w/o aura maps to mwoa
3964735629103, migraine w/o aura maps to mwoa
4166259653173, migraine w/o aura maps to mwoa
6925948962043, migraine w/o aura maps to mwoa
7277988854713, migraine w/o aura maps to mwoa
9041302068919, migraine w/o aura maps to mwoa
53843667737173, migraine w/o aura maps to mwoa
56580493999543, migraine w/o aura maps to mwoa
70288832213209, migraine w/o aura maps to mwoa
122786715194029, migraine w/o aura maps to mwoa
1669153699852363, migraine w/o aura maps to mwoa
1753995313985833, migraine w/o aura maps to mwoa
2178953798609479, migraine w/o aura maps to mwoa
3806388171014899, migraine w/o aura maps to mwoa
29591598361760989, migraine w/o aura maps to mwoa
917339549214590659, migraine w/o aura maps to mwoa
1145520433,ndph maps to ndph
2106413,exercise maps to exercise
17411171,thunderclap maps to thunderclap
4026427,coldHA maps to coldHA
1069111207,compression maps to compression
1183350401,traction maps to traction
1577982193416431,cluster maps to cluster
1932584933510011,cluster maps to cluster
2251727399594233,cluster maps to cluster
3492836989921763,cluster maps to cluster
4237502744118281,cluster maps to cluster
4911247950296083,cluster maps to cluster
7428927404960501,cluster maps to cluster
7960831515100871,cluster maps to cluster
8173593159157019,cluster maps to cluster
699200435231955421,ph maps to ph
856324128542507201,ph maps to ph
997735452522003803,ph maps to ph
1547668379108935033,ph maps to ph
1877628135061093771,ph maps to ph
2176163152351142153,ph maps to ph
3291741374856059791,ph maps to ph
3527426914821887461,ph maps to ph
3621701130808218529,ph maps to ph
444401003390335,sun maps to sun
544266397410635,sun maps to sun
634145252028905,sun maps to sun
983674131099955,sun maps to sun
1193391458542585,sun maps to sun
1383135707181155,sun maps to sun
2092180004725285,sun maps to sun
2301897332167915,sun maps to sun
54659995058027,hc maps to hc
66943140014887,hc maps to hc
77997970476061,hc maps to hc
120988977825071,hc maps to hc
146783582234477,hc maps to hc
170121557652511,hc maps to hc
257331886846217,hc maps to hc
275756604281507,hc maps to hc
283126491255623,hc maps to hc
75121389451,mwa maps to mwa
179941932871,mwa maps to mwa
188719588133,mwa maps to mwa

209121705769,mwa maps to mwa
345049093919,mwa maps to mwa
581872457273,mwa maps to mwa
594604896163,mwa maps to mwa
635348700611,mwa maps to mwa
716836309507,mwa maps to mwa
826512945899,mwa maps to mwa
866830650577,mwa maps to mwa
889227610013,mwa maps to mwa
910191718697,mwa maps to mwa
932604566599,mwa maps to mwa
954591314731,mwa maps to mwa
960542072261,mwa maps to mwa
1033426681907,mwa maps to mwa
1057790375783,mwa maps to mwa
1393787513933,mwa maps to mwa
1424286146623,mwa maps to mwa
1461777148759,mwa maps to mwa
1493763519629,mwa maps to mwa
1521881771231,mwa maps to mwa
1596119906413,mwa maps to mwa
1619807110787,mwa maps to mwa
1655251467697,mwa maps to mwa
1717073020447,mwa maps to mwa
1768673409809,mwa maps to mwa
1800832679981,mwa maps to mwa
1995517294033,mwa maps to mwa
2233913264179,mwa maps to mwa
2286579195751,mwa maps to mwa
2475417400847,mwa maps to mwa
2533776946643,mwa maps to mwa
2596169469181,mwa maps to mwa
2657375822089,mwa maps to mwa
4084418344297,mwa maps to mwa
4180711114693,mwa maps to mwa
4283658263531,mwa maps to mwa
4384648242239,mwa maps to mwa
4746756454183,mwa maps to mwa
4858664268427,mwa maps to mwa
6887746063999,mwa maps to mwa
7038462608069,mwa maps to mwa
7050129075331,mwa maps to mwa
7204398858161,mwa maps to mwa
7223733676877,mwa maps to mwa
7381802247487,mwa maps to mwa
7394037810713,mwa maps to mwa
7520755549093,mwa maps to mwa
7555832948803,mwa maps to mwa
7698062163217,mwa maps to mwa
7887621673439,mwa maps to mwa
8004677858161,mwa maps to mwa
8073577390691,mwa maps to mwa
8179834922891,mwa maps to mwa
8193393249709,mwa maps to mwa
8372679754079,mwa maps to mwa
8485341431141,mwa maps to mwa
8685388773329,mwa maps to mwa
8740337530027,mwa maps to mwa
8899260525343,mwa maps to mwa
8946396568063,mwa maps to mwa
9109066274467,mwa maps to mwa
9861342744299,mwa maps to mwa
10093830196031,mwa maps to mwa
10260855840551,mwa maps to mwa
10502762068619,mwa maps to mwa
11299697190653,mwa maps to mwa
11370137553043,mwa maps to mwa
11638195805767,mwa maps to mwa
11924778409289,mwa maps to mwa

12205912674341,mwa maps to mwa
12521286076129,mwa maps to mwa
13132080518867,mwa maps to mwa
17303362063217,mwa maps to mwa
17681991430027,mwa maps to mwa
17711299872173,mwa maps to mwa
18098855668063,mwa maps to mwa
18893605403819,mwa maps to mwa
19173995799781,mwa maps to mwa
19339034214911,mwa maps to mwa
19593558071111,mwa maps to mwa
19626034993489,mwa maps to mwa
20055488713259,mwa maps to mwa
20109312668063,mwa maps to mwa
20549341391653,mwa maps to mwa
20583402554147,mwa maps to mwa
20936157339367,mwa maps to mwa
21033805235857,mwa maps to mwa
21316833351403,mwa maps to mwa
21429740616523,mwa maps to mwa
21819391308607,mwa maps to mwa
21957433307141,mwa maps to mwa
22475093817329,mwa maps to mwa
23621355875879,mwa maps to mwa
24178244423051,mwa maps to mwa
24773617138117,mwa maps to mwa
25357670980273,mwa maps to mwa
31455913801007,mwa maps to mwa
51901998960457,mwa maps to mwa
57513025875101,mwa maps to mwa
60318539332423,mwa maps to mwa
87524773154719,mwa maps to mwa
89439976068389,mwa maps to mwa
95568625392133,mwa maps to mwa
96986910793067,mwa maps to mwa
99109162670377,mwa maps to mwa
101717979612241,mwa maps to mwa
103943755971371,mwa maps to mwa
105900368677769,mwa maps to mwa
107825924039621,mwa maps to mwa
111066240320587,mwa maps to mwa
119482780692553,mwa maps to mwa
125311209019019,mwa maps to mwa
144483943052083,mwa maps to mwa
243650044187461,mwa maps to mwa
248981555001191,mwa maps to mwa
266042389605127,mwa maps to mwa
300164058812999,mwa maps to mwa
613582,sex maps to sex
3829598,sex maps to sex
167814677,sex maps to sex
1047395053,sex maps to sex
14937586453,stabbing maps to stabbing
15465416363,stabbing maps to stabbing
16204378237,stabbing maps to stabbing
16415510201,stabbing maps to stabbing
16521076183,stabbing maps to stabbing
16732208147,stabbing maps to stabbing
18632395823,stabbing maps to stabbing
19371357697,stabbing maps to stabbing
331231589,nummular maps to nummular
14155130507,hypnic maps to hypnic
14655311797,hypnic maps to hypnic
15355565603,hypnic maps to hypnic
17956508311,hypnic maps to hypnic
15555638119,hypnic maps to hypnic
15655674377,hypnic maps to hypnic
15855746893,hypnic maps to hypnic
17656399537,hypnic maps to hypnic

18356653343,hypnic maps to hypnic
1412526109,cough maps to cough
1440706181,cough maps to cough
1378253359942681,infrequent tension type headache maps to itth
1386197183342927,infrequent tension type headache maps to itth
2206388426002661,infrequent tension type headache maps to itth
2219105362175587,infrequent tension type headache maps to itth
2241879928833803,infrequent tension type headache maps to itth
2254801426982701,infrequent tension type headache maps to itth
9699783080351321,infrequent tension type headache maps to itth
9755689611073807,infrequent tension type headache maps to itth
9855811762608983,infrequent tension type headache maps to itth
9912617594093761,infrequent tension type headache maps to itth
15777758744434123,infrequent tension type headache maps to itth
15868696835180141,infrequent tension type headache maps to itth
514088503258620013,infrequent tension type headache maps to itth
517051549386911771,infrequent tension type headache maps to itth
522358023418276099,infrequent tension type headache maps to itth
525368732486969333,infrequent tension type headache maps to itth
836221213455008519,infrequent tension type headache maps to itth
841040932264547473,infrequent tension type headache maps to itth
3676217787453150659,infrequent tension type headache maps to itth
3697406362596972853,infrequent tension type headache maps to itth
194839542735016984927,infrequent tension type headache maps to itth
195962537217639561209,infrequent tension type headache maps to itth
21262771073163,frequent tension type headache maps to ftth
21385323067821,frequent tension type headache maps to ftth
34038685022703,frequent tension type headache maps to ftth
34234873409001,frequent tension type headache maps to ftth
34586224191969,frequent tension type headache maps to ftth
34785568423623,frequent tension type headache maps to ftth
149641766231883,frequent tension type headache maps to ftth
150504254798061,frequent tension type headache maps to ftth
152048872391109,frequent tension type headache maps to ftth
152925234768003,frequent tension type headache maps to ftth
243408709879329,frequent tension type headache maps to ftth
244811641924743,frequent tension type headache maps to ftth
7931013610289799,frequent tension type headache maps to ftth
7976725504297233,frequent tension type headache maps to ftth
8058590236728777,frequent tension type headache maps to ftth
8105037442704159,frequent tension type headache maps to ftth
12900661623604437,frequent tension type headache maps to ftth
12975017022011379,frequent tension type headache maps to ftth
56714229401883657,frequent tension type headache maps to ftth
57041112568465119,frequent tension type headache maps to ftth
3005854158299833821,frequent tension type headache maps to ftth
3023178966128651307,frequent tension type headache maps to ftth
5029577936481697,chronic tension type headache maps to ctth
5058566858305799,chronic tension type headache maps to ctth
8051641932650957,chronic tension type headache maps to ctth
8098049090764219,chronic tension type headache maps to ctth
8181158961058211,chronic tension type headache maps to ctth
8228312615012437,chronic tension type headache maps to ctth
9281386088971379,chronic tension type headache maps to ctth
9334881109657093,chronic tension type headache maps to ctth
14858184597366199,chronic tension type headache maps to ctth
14943822548936033,chronic tension type headache maps to ctth
15097190247725977,chronic tension type headache maps to ctth
15184205753476559,chronic tension type headache maps to ctth
35396840949201377,chronic tension type headache maps to ctth
35600857323548359,chronic tension type headache maps to ctth
35966227130689871,chronic tension type headache maps to ctth
36173525269771657,chronic tension type headache maps to ctth
57576835707070051,chronic tension type headache maps to ctth
57908690667917717,chronic tension type headache maps to ctth
65319943607289139,chronic tension type headache maps to ctth
65696427432115013,chronic tension type headache maps to ctth
66370666560757597,chronic tension type headache maps to ctth
66753206425661099,chronic tension type headache maps to ctth

106250037026448857,chronic tension type headache maps to ctth
106862429170693519,chronic tension type headache maps to ctth
1876032570307672981,chronic tension type headache maps to ctth
1886845438148063027,chronic tension type headache maps to ctth
1906210037926563163,chronic tension type headache maps to ctth
1917196839297897821,chronic tension type headache maps to ctth
3051572292474712703,chronic tension type headache maps to ctth
3069160605399639001,chronic tension type headache maps to ctth
3461957011186324367,chronic tension type headache maps to ctth
3481910653902095689,chronic tension type headache maps to ctth
3517645327720152641,chronic tension type headache maps to ctth
3537919940560038247,chronic tension type headache maps to ctth
5631251962401789421,chronic tension type headache maps to ctth
5663708746046756507,chronic tension type headache maps to ctth
13415402719747321883,chronic tension type headache maps to ctth
13492724925624828061,chronic tension type headache maps to ctth
24756258627162583681,chronic tension type headache maps to ctth
24898945996771589927,chronic tension type headache maps to ctth
711016344146608059799,chronic tension type headache maps to ctth
715114421058115887233,chronic tension type headache maps to ctth
1312081707239616935093,chronic tension type headache maps to ctth
1319644137828894266131,chronic tension type headache maps to ctth
